# Case Report: Hyperammonemic Encephalopathy Linked to *Ureaplasma* spp. and/or *Mycoplasma hominis* Systemic Infection in Patients Treated for Leukemia, an Emergency Not to Be Missed

**DOI:** 10.3389/fonc.2022.912695

**Published:** 2022-07-08

**Authors:** Manon Delafoy, Juliette Goutines, Aude-Marie Fourmont, André Birgy, Maryline Chomton, Michaël Levy, Jérôme Naudin, Lara Zafrani, Lou Le Mouel, Karima Yakouben, Aurélie Cointe, Marion Caseris, Matthieu Lafaurie, Stéphane Bonacorsi, Françoise Mechinaud, Sabine Pereyre, Nicolas Boissel, André Baruchel

**Affiliations:** ^1^ Department of Hematology, Robert Debré University Hospital, Assistance Publique-Hôpitaux de Paris, Université Paris Cité, Paris, France; ^2^ Department of General Pediatrics, Pediatric Infectious Disease and Internal Medicine, Robert Debré University Hospital, Assistance Publique-Hôpitaux de Paris, Université Paris Cité, Paris, France; ^3^ Department of Hematology, Saint-Louis University Hospital, Assistance Publique-Hôpitaux de Paris, Université Paris Cité, Paris, France; ^4^ Department of Microbiology, Robert-Debré Hospital, Assistance Publique-Hôpitaux de Paris, Université Paris Cité, Paris, France; ^5^ Infection, Antimicrobiens, Modélisation, Evolution, Unité Mixte de Recherche 1137, Institut National de la Santé Et de la Recherche Médicale, Université Paris Cité, Paris, France; ^6^ Paediatric Intensive Care Unit, Robert-Debré University Hospital, Assistance Publique-Hôpitaux de Paris, Université Paris Cité, Paris, France; ^7^ Medical Intensive Care Unit, Saint-Louis University Hospital, Assistance Publique-Hôpitaux de Paris, Université Paris Cité, Paris, France; ^8^ Unité Mixte de Recherche 976, Institut National de la Santé Et de la Recherche Médicale, Paris, France; ^9^ Department of Infectious Diseases, Saint-Louis University Hospital, Assistance Publique-Hôpitaux de Paris, Université Paris Cité, Paris, France; ^10^ Department of Bacteriology, National Reference Center for Bacterial Sexually Transmitted Infections, Bordeaux University Hospital, Bordeaux, France; ^11^ Unité Mixte de Recherche 5234, Fundamental Microbiology and Pathogenicity, Université de Bordeaux, Centre National de la Recherche Scientifique, Bordeaux, France; ^12^ Research Unit EA-3518, Université Paris Cité, Paris, France

**Keywords:** *Ureaplasma* spp., *Mycoplasma* spp., systemic infection, hyperammonemic encephalopathy, immunocompromised patients, case report

## Abstract

**Background:**

Hyperammonemic encephalopathy caused by *Ureaplasma* spp. and *Mycoplasma hominis* infection has been reported in immunocompromised patients undergoing lung transplant, but data are scarce in patients with hematological malignancies.

**Case Presentation:**

We describe the cases of 3 female patients aged 11–16 years old, developing initially mild neurologic symptoms, rapidly evolving to coma and associated with very high ammonia levels, while undergoing intensive treatment for acute leukemia (chemotherapy: 2 and hematopoietic stem cell transplant: 1). Brain imaging displayed cerebral edema and/or microbleeding. Electroencephalograms showed diffuse slowing patterns. One patient had moderate renal failure. Extensive liver and metabolic functions were all normal. *Ureaplasma* spp. and *M. hominis* were detected by PCR and specific culture in two patients, resulting in prompt initiation of combined antibiotics therapy by fluoroquinolones and macrolides. For these 2 patients, the improvement of the neurological status and ammonia levels were observed within 96 h, without any long-term sequelae. *M. hominis* was detected post-mortem in vagina, using 16S rRNA PCR for the third patient who died of cerebral edema.

**Conclusion:**

Hyperammonemic encephalopathy linked to *Ureaplasma* spp. and *M. hominis* is a rare complication encountered in immunocompromised patients treated for acute leukemia, which can lead to death if unrecognized. Combining our experience with the few published cases (n=4), we observed a strong trend among female patients and very high levels of ammonia, consistently uncontrolled by classical measures (ammonia-scavenging agents and/or continuous kidney replacement therapy). The reversibility of the encephalopathy without sequelae is possible with prompt diagnosis and adequate combined specific antibiotherapy. Any neurological symptoms in an immunocompromised host should lead to the measurement of ammonia levels. If increased, and in the absence of an obvious cause, it should prompt to perform a search for *Ureaplasma* spp. and *M. hominis* by PCR as well as an immediate empirical initiation of combined specific antibiotherapy.

## Introduction

Hyperammonemic encephalopathy (HE) is a rare and often lethal complication of intensive chemotherapy or hematopoietic stem cell transplantation (HSCT), in patients treated for hematologic pathologies. It was first described by Watson et al. in 1985, as “transient idiopathic hyperammonemia (IHA)” (1), in patients without any liver nor metabolic disorder. There are few data regarding its occurrence. Davies et al. published in 1996 the largest series of adult patients undergoing HSCT, and presenting with IHA, with an incidence of 0.5% (12/2,358 patients) and a mortality rate of 83% (10/12 patients) (2). Most common symptoms are those of a metabolic encephalopathy, with abrupt alteration in the mental status, sometimes seizures, confusion, and lethargy, leading to coma. The diagnosis is often made when severe neurologic symptoms occur, in the absence of liver or metabolism impairment. Despite ammonia-scavenging drugs associated with dialysis, the prognosis remains poor.

Several causes of IHA have been proposed during the last decades, such as iatrogenic toxicity (chemotherapy (3, 4), steroids, supportive care medications) and an increased catabolic state during acute severe illness. Recently, *U. parvum* and *U. urealyticum* infections have been determined to cause hyperammonemia in adult lung transplant recipients, responsible for numerous neurological manifestations (5). This infectious hypothesis has been later described in three patients treated for acute myeloblastic leukemia (AML), aged 21, 16, and 12 years old, following HSCT or not, with prompt resolution of hyperammonemic encephalopathy after the initiation of appropriate antibiotic therapy (6–8). Recently, Tawfik et al. described the case of a 53-year-old patient, receiving chimeric receptor antigen T-cell (CAR-T) therapy for relapsing B acute lymphoblastic leukemia (B-ALL), who died of brain damage following a systemic *U. parvum* infection (9).

Here, we report the case of 3 immunocompromised patients treated for acute leukemia, aged 11–16 years old, diagnosed with systemic *Ureaplasma* spp. or *M. hominis* infections, responsible for HE. Pooling these cases to the ones described in the literature allowed us to make some recommendations for the diagnosis and treatment of this misleading, rare, and severe condition.

## Case Presentation

During these last three years (January 2019–December 2021), 361 patients were treated for a newly diagnosed acute leukemia in the pediatric hematology department of the Hôpital Universitaire Robert Debré (Paris, France) and the adolescent/young adult unit of the Hôpital Universitaire Saint Louis (Paris, France). We report here on 3 patients presenting with HE observed while undergoing intensive treatment for B-ALL (n=1) or AML (n=2), corresponding to an incidence of 0.8%. Clinical, main biological, and imaging feature characteristics are detailed in **Table 1** and treatment/outcome in **Table 2**.

**Table 1:** Clinical, biological, and imaging characteristics at onset of the three newly reported patients.

	Patient #1	Patient #2	Patient #3
**Clinical characteristics**			
Age at time of complication (years)	15	11	16
Gender	Female	Female	Female
Disease	B-ALL	AML	AML
Intensive chemotherapy	No	Yes	Yes
HSCT	Yes	No	No
Aplasia ongoing at the time of infection	Yes	Yes	Yes
Fever ongoing at time of complication	No	Yes	Yes
Perineal complication ongoing at time of complication	Yes (hematocolpos)	Yes (acute urinary retention)	Yes (pelvic cellulitis)
Initial neurological symptoms	Irritability/confusion/seizure	Irritability/confusion	Psychomotor slowing
Day of onset	*20*	*24*	*19*
Secondary neurological symptoms	Coma	Coma	Coma
GCS score	8	6	12
**Biology**			
NH4 level max in µmol/L (normal range)	533 (14-38)	1,420 (14-38)	656 (18-72)
CRP max (mg/L)	287	410	276
Liver biology	Bilirubin 1.5N	ASAT/ALAT 2.5N Gamma GT 17N Bilirubin normal	Normal
**Imaging**			
Liver imaging (normal/abnormal)	Normal	Normal	NA
EEG abnormality	Yes (poorly responsive)	Yes (poorly responsive)	Yes (poorly responsive)
Cerebral Imaging	MRI	MRI	CT scan
*Abnormalities*	Yes (bilateral cerebral edema with a right frontal microbleeding)	Yes (left cerebellar microbleeds with hemorrhagic sequelae)	Yes (cerebral edema)
**Bacterial documentation**			
Species	*U. parvum*, *U. urealyticum*, *M. hominis*	*U. parvum*, *M. hominis*	*M. hominis*
*Method*	PCR (vagina, blood, urine, trachea) and culture (trachea)	PCR (vagina, blood, urine) and culture (blood)	16s rRNA PCR (vagina)

B-ALL, B acute lymphoid leukemia; AML, acute myeloid leukemia; HSCT, hematopoietic stem cell transplant; GCS score, Glasgow Coma Scale score; NA, not applicable; EEG, electroencephalogram; MRI, magnetic resonance imaging; CT scan, computed tomography scan; U, ureaplasma; M, mycoplasma.

**Table 2:** Therapeutics and outcome of the 3 newly reported patients.

	Patient #1	Patient #2	Patient #3
**Treatment**			
Antibiotherapy	Levofloxacin + azithromycin	Levofloxacin + clarithromycin switched to josamycin	None
NH_4_ chelation*	Yes	Yes	Yes
Renal replacement therapy	Yes	Yes	Yes
**Outcome**			
Alive	Yes	Yes	No
*Cause of death*	*NA*	*NA*	*Cerebral edema*
*Neurological sequelae*	*No*	*No*	*NA*
*Follow-up (year)*	1.5	0.8	*NA*

*NH_4_ chelation: sodium phenylbutyrate and/or sodium benzoate.

NA, not applicable.

### Patient #1

**Figure 1:**
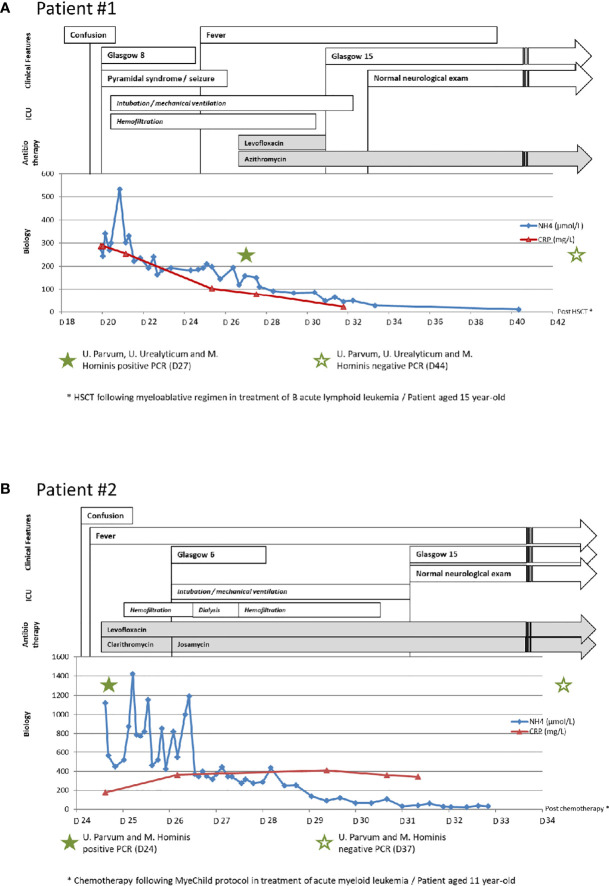
Clinical, biological, and imaging features and treatment of patients. **(A)** Clinical, biological, and imaging features and treatment of patient #1. **(B)** Clinical, biological, and imaging features and treatment of patient #2. HSCT, hematopoietic stem cell transplant.

A 15-year-old female patient treated for B-ALL underwent haplo-identical HSCT following a myeloablative conditioning regimen in first remission. From day 6 post-HSCT, she presented with refractory thrombocytopenia responsible for hematocolpos (blood-filled dilated vagina). Opioids were started for a grade IV mucositis, complicated by iatrogenic acute urinary retention requiring indwelling catheter between day 11 and day 15 post-HSCT. On day 20, mental status changes were observed, shortly leading to coma [Glasgow Coma Scale (GCS) score of 8] followed by seizure and pyramidal syndrome. The brain computerized tomography (CT) scan was normal. Routine biological exams showed acute kidney injury (creatinine level at 142 µmol/L), a mildly elevated total bilirubin level (38 µmol/L), inflammatory markers (CRP level 287 mg/L), and a highly increased ammonia level at 342 µmol/L (normal 14-38 µmol/L). Ammonia-scavenging drugs were immediately started. The patient was then transferred to the intensive care unit (ICU) and was intubated for airway protection and placed on continuous venovenous hemofiltration to decrease ammonia levels. Other extensive liver and metabolic tests were normal. Ammonia levels plateaued for 6 days after ICU admission, and her neurological state worsened with abnormal respiratory patterns and the absence of corneal reflex. The MRI performed on day 25 post-HSCT showed a bilateral cerebral edema with a right frontal microbleeding. Electroencephalograms (EEGs) showed diffuse slowing patterns. On day 27 post-HSCT, blood, tracheal, urine, and vaginal samples were sent for *Ureaplasma* spp./*M. hominis*-specific culture and PCR. Empiric antibiotics (levofloxacin and azithromycin) were started on the same day. Within 48 h, her neurological status improved and ammonia levels normalized, allowing to stop life-sustaining therapies in the ICU. PCR was positive for *U. parvum* (blood, trachea, urine, and vagina), *U. urealyticum* (urine and vagina), and *M. hominis* (urine, vagina, and trachea). The latter was also positive in the culture of tracheal aspiration, allowing the determination of antibiotic susceptibility. *Ureaplasma* spp. were susceptible to erythromycin, tetracycline, levofloxacin, and moxifloxacin, with intrinsic resistance to clindamycin*. M. hominis*, intrinsically resistant to erythromycin and azithromycin, was susceptible to tetracycline, clindamycin, and moxifloxacin but had acquired resistance to levofloxacin. Levofloxacin and azithromycin were discontinued 4 and 20 days later, respectively, with a strict monitoring of neurological examination and ammonia levels, which both remained normal. Eighteen months post-HSCT, the patient is still in remission, with a normal mental status and neurological exam. Clinical, biological, and imaging features are summarized in **Figure 1A**.

### Patient #2

An 11-year-old female patient was diagnosed with AML, presenting with cauda equina syndrome. Given the neurological presentation, steroids and chemotherapy were started promptly but dysuria remained a major issue, requiring an indwelling urinary catheter. On day 24 of chemotherapy, she developed mental status changes with irritability and confusion (GCS score of 14). The brain CT scan was normal. Routine biological exams revealed mild anicteric cholestasis (elevated gamma GT up to 407 UI/L with normal bilirubin levels), inflammatory markers (CRP level 179 mg/L), and extremely high ammonia levels at 1,118 µmol/L (normal 14-38 µmol/L). Given the previous case observed earlier, samples for the *Ureaplasma* spp.*/M. hominis-*specific culture and PCR were promptly performed. Empirical antibiotherapy combining levofloxacin and clarithromycin was immediately started. PCR performed on urine, vaginal, and blood samples was positive for *U. parvum* and *M. hominis.* The latter was also detected in blood culture. Antibiogram showed susceptibility to tetracycline, clindamycin, levofloxacin, and moxifloxacin and intrinsic resistance to erythromycin. Other extensive liver and metabolic tests were normal. The patient was transferred to the ICU and placed under continuous venovenous hemofiltration, with ammonia-scavenging drugs. Despite 48 h of intensive treatment, ammonia levels remained elevated and the neurological examination worsened, leading to a coma (GCS score of 6) with EEG showing diffuse slowing patterns. She was intubated the same day and benefited from two intermittent hemodialysis sessions. Clarithromycin was switched to josamycin. Within 72 h of newly combined antibiotics, ammonia levels started to decrease, and her mental status improved, allowing to stop life-sustaining therapies a few days later. MRI performed on day 32 post-chemotherapy showed left cerebellar microbleeds with hemorrhagic sequelae. Josamycin and levofloxacin were stopped after 21 and 47 days, respectively, with a strict monitoring of ammonia levels and neurological examination, which both remained normal. After 10 months of AML treatment, the patient is in remission without any sequelae. Clinical, biological, and imaging features are summarized in **Figure 1B**.

### Patient #3

A 16-year-old female patient was diagnosed with AML and received leukoreductive therapy followed by conventional chemotherapy. On day 9 post-chemotherapy, she presented sepsis while being profoundly neutropenic and was started on empirical antibiotics (cefepime plus vancomycin). No detectable microorganism was documented in repeated blood cultures and local samplings. Despite broad spectrum antibiotics (meropenem, amikacine and ornidazole) the infection kept on progressing with perineal cellulitis, the persistence of major hyperthermia, and biological inflammatory markers. On day 19 post-chemotherapy, she developed psychomotor slowing. Routine biological examinations were normal except for elevated CRP (271 mg/L), and the brain CT scan showed no abnormalities. On day 22 post-chemotherapy, the neurological examination worsened with dizziness and confusion (GCS score of 12), leading to a transfer in ICU for mechanical ventilation. On day 23, she presented seizure with anisocoria, revealing major cerebral edema on a brain CT scan together with hyperammonemia up to 530 µmol/L (normal 18-72µmol/L). Hemodialysis and the intake of ammonia-scavenging drugs were promptly begun, but her neurological condition worsened within 24 h, leading to brain death. Extensive liver and metabolic tests proved normal. Post-mortem, *M. hominis* was identified using 16S rRNA PCR in a vaginal sample, an infection for which the patient has not received adapted antibiotics.

## Discussion

IHA occurring in patients treated for malignancies, while receiving intensive chemotherapy or HSCT, has been described over the last decades but with difficulties to identify one predominant etiology (3, 4). Evidence came from immunocompromised patients post-lung transplant who experienced HE associated with *M. hominis* or *Ureaplasma* spp. infections (10, 11). In a recent meta-analysis (11), *Ureaplasma* spp.–infected lung transplant recipients showed a higher incidence of hyperammonemia syndrome and peak ammonia concentration compared with *other* recipients (41.67% vs. 2.84%), with an increased risk of 14.64 (CI: 2.85–75.24).

We describe here the cases of 3 patients, treated for hematologic malignancies with intensive therapy (chemotherapy or HSCT), presenting HE secondary to *M. hominis* or *Ureaplasma* spp. infections. All 3 patients were females, pre-pubescent or pubescent, with urogenital complications occurring while being profoundly neutropenic. The first symptoms of HE began around day 20 after chemotherapy initiation or HSCT, quickly leading to severe neurological symptoms without major liver or metabolic complications. When *M. hominis* or *Ureaplasma* spp. infections were diagnosed and treated, their neurological state quickly returned to normal (<96 h), without any sequalae, even with extremely high ammonia levels and worrying cerebral imaging. Ammonia-scavenging drugs and renal replacement therapies seemed insufficient to improve a patient’s clinical status.

Only 4 similar cases in patients treated with intense therapy and immunosuppression for hematologic malignancies have been recently described in the literature (**Table 3**) (6–9). We report here the second case during an HSCT procedure. Combined with our 3 cases, they show a strong trend among female (6 females vs. 1 male) and pediatric patients (age 11–21 years old, n=6). No other pelvic/perineal complication is described. *Ureaplasma* spp. was more frequently involved (n=6), and all microbiologic documentations were performed by PCR. Once an infection is suspected and/or documented, empiric antibiotherapy combining fluoroquinolones with macrolide or tetracycline was used, with the normalization of ammonia levels and neurological status in 5 out of 6 patients. The last patient was a 53-year-old woman treated with CAR T-cell therapy for B-ALL. She developed HE while under levofloxacin prophylaxis. Once elevated ammonia levels were identified, doxycycline was introduced to cover ammonia-producing bacteria without any clinical or biological efficacy. PCR from broncho-alveolar lavage returned positive for *U. parvum*, and levofloxacin was started again, as a combined targeted antibiotherapy. The cerebral MRI performed the same day showed diffuse cerebral edema, and the patient’s family elected to the discontinuation of care (9).

**Table 3:** Comparison of cases of hyperammonemic encephalopathy due to *Ureaplasma* spp. and/or *Mycoplasma hominis* in immunocompromised patients treated for a malignant hemopathy found in the literature.

	Age (Y)	Gender	Disease/Treatment	Perineal Complication	Bacteria	Method of Detection	Antibiotics	Outcome
This Study	15 11 16	Female Female Female	B-ALL/HSCT AML/CT AML/CT	Yes Yes Yes	*U. parvum, U. urealyticum, M. hominis* *U. parvum, M. hominis* *M. hominis*	PCR (vagina, urine, blood, trachea) and culture (trachea) PCR (vagina, urine, blood) and culture (blood) Post-mortem 16sRNA PCR (vagina)	Levofloxacin + azithromycin Levofloxacin + clarythromycin/josamycin None	Alive Alive Dead (cerebral edema)
Placone et al. (8)	16	Female	AML/CT	No	*U. parvum*	PCR (blood)	Azithromycin then doxycycline	Alive
Smith et al. (7)	12	Female	AML/CT	No	*U. parvum*	PCR (blood, urine, and trachea) Culture failure	Doxycycline then azithromycin + levofloxacin/moxifloxacin	DOD/normalization of NH4 level
Graetz et al. (6)	21	Male	AML/HSCT	No	*U. parvum*	PCR (trachea)	Azithromycin + levofloxacin	Alive
Tawfik et al. (9)	53	Female	B-ALL/CAR T cells	No	*U. parvum*	PCR (BAL fluid)	Doxycycline (D1) + levofloxacin (D5)	Dead (discontinuation of care due to the severity of the brain damage)

Y, years; B-ALL, B acute lymphoid leukemia; HSCT, hematopoietic stem cell transplant; U, ureaplasma; M, mycoplasma; AML, acute myeloid leukemia; CT, chemotherapy; DOD, died of disease; CAR-T cells, chimeric antigen receptor-modified T cells; BAL fluid, bronchoalveolar lavage fluid.

HE linked to *Ureaplasma* spp. or *M. hominis* systemic infection is rarely reported in literature and may be underestimated. In a recent study, Zaho et al. (12) published a series of 265 adults treated in ICU for sepsis, 107 of whom had non-hepatic hyperammonemia. They compared the characteristics of this group to the non-hyperammonemia group. The median white blood cell count was similar for both groups and well above bone marrow aplasia criteria. Patients with non-hepatic hyperammonemia had more intestinal and urinary tract infections (23.4% vs. 13.3%, p=0.034 and 45.8% vs. 24.7%, p<0.001, respectively) and had a higher rate of encephalopathy (37.4% vs. 19.6%, p=0.001) and hospital mortality (59.8% vs. 43%, p=0.007). Infection by *Escherichia coli* was found in 42.1% of patients in this group (vs. 22.8%, p=0.001). *Mycoplasma* and *Ureaplasma* spp. were not described in this study, but these microorganisms are known to require a specific growth medium, making their identification more difficult. Molecular methods like PCR have been developed to increase the detection sensitivity, but these diagnostic tools may be only available in specialized laboratories (13). Moreover, the delay required to complete a microbiological diagnosis is often incompatible with the severity of symptoms occurring in immunocompromised patients. Thus, the general suggestion is to probabilistically combine two molecules active on *M. hominis* and *Ureaplasma* spp. in the case of severe infections (14, 15).

The best antibiotic therapy strategy against *M. hominis* or *Ureaplasma* spp. is yet to be defined in these situations. Since they lack peptidoglycan, these bacteria are naturally resistant to beta lactams. Lincosamides are ineffective on *Ureaplasma* spp., while *M. hominis* is intrinsically resistant to 14- and 15-membered macrolides like erythromycin and azithromycin but remains susceptible to josamycin (16–18). Therefore, the recommended molecules are fluoroquinolones, macrolides (josamycin), and tetracyclines. Among the fluoroquinolones, moxifloxacin has the lowest minimal inhibitory concentrations (19), but given its potential effect on the QT interval, the use of levofloxacin should be preferred as a first-line treatment. Resistance has nevertheless been described for each of these antibiotic classes. For example, the most recent French study on the general population reported a levofloxacin resistance rate of 1.2% and 2.7% and tetracycline resistance rate of 14.8% and 7.5% for *Ureaplasma* spp. and *M. hominis*, respectively (19). For patient #1, *M. hominis* had an acquired resistance to levofloxacin. Nevertheless, given the clinical and biological improvements, *Ureaplasma* spp. was most likely to be responsible for HE and initial antibiotherapy was not adapted. For patient #2, both microorganisms were positive by PCR but only *M. hominis* was identified on a specific culture and found sensitive to levofloxacin but resistant to 14- and 15-membered macrolides. Given the absence of rapid clinical and biological improvements, we decided to keep an antibiotherapy combining fluroquinolone and macrolide in order to cover rare levofloxacin-resistant *Ureaplasma* spp. Clarithromycin was switched to josamycin, a 16-membered macrolide effective for both germs (no occurring macrolide-resistant *U. parvum* has been identified in France) (20).

Treatment duration is another challenging question for these patients undergoing severe neutropenia. For patient #1, antibiotic therapy was stopped after hematological recovery and 20 days of treatment. For patient #2, who developed severe hematuria, requiring numerous surgical interventions, the use of josamycin and levofloxacin was respectively maintained to 18 and 45 days after HE recovery.

Considering the low incidence (0.5%–0.8%) but severity of this complication, a screening for *Ureaplasma* spp. and *M. hominis* could be discussed in immunocompromised patients treated for a malignant hemopathy. *Ureaplasma* spp. is found in 40%–80% of healthy adult women’s genital flora (21), whereas 21%–53% of sexually active women are colonized with *M. hominis* (22); both bacteria are less diagnosed in men. The colonization rate is maximal at puberty and is linked to a low socio-economic level, oral contraception, sexual activity, and multiple partners (22). Prospective studies on patients experiencing HE while undergoing intensive treatment for a malignant hemopathy should allow a better estimate of its incidence. If higher than described in literature, it should be interesting to discuss a systematic screening in this population, especially in young women, with urogenital complications.

In conclusion, HE due to *Ureaplasma* spp. and *M. hominis* infection in patients treated for acute leukemia is a rare but possibly underestimated complication that can lead to death if not recognized and managed promptly. Thus, in this population, any neurological symptoms should lead to the measurement of ammonia levels. If increased, and in the absence of an obvious cause, this should prompt to search for *Ureaplasma* spp. and *M. hominis* by PCR in various sites (vagina, urine, blood, and trachea if possible). Immediate empiric antibiotic therapy combining fluoroquinolones (levofloxacin) and macrolides (josamycin) or tetracycline for older patients should be initiated. Supportive care in ICU is required, including ammonia-scavenging drugs and potential kidney replacement therapies. Appropriate antibiotics seem to allow a rapid and complete recovery in 72-96 h, even when ammonia levels are very high and brain imaging worrying.

## Data Availability Statement

The raw data supporting the conclusions of this article will be made available by the authors, without undue reservation.

## Ethics Statement

The studies involving human participants were reviewed and approved by Comité Local d’Ethique pour la Recherche Clinique des HUPSSD Avicenne-Jean Verdier-René Muret. Written informed consent to participate in this study was provided by the participants’ legal guardian/next of kin.

## Author Contributions

MD and AB contributed to the conception of the study, the interpretation of data, and manuscript writing. JG and AMF wrote sections of the manuscript. All authors contributed to the collection and assembly of data and the final approval of the submitted version.

## Conflict of Interest

The authors declare that the research was conducted in the absence of any commercial or financial relationships that could be construed as a potential conflict of interest.

## Publisher’s Note

All claims expressed in this article are solely those of the authors and do not necessarily represent those of their affiliated organizations, or those of the publisher, the editors and the reviewers. Any product that may be evaluated in this article, or claim that may be made by its manufacturer, is not guaranteed or endorsed by the publisher.
